# MicroRNA expression profiles of bovine monocyte-derived macrophages infected in vitro with two strains of *Streptococcus agalactiae*

**DOI:** 10.1186/s12864-018-4591-3

**Published:** 2018-04-10

**Authors:** Anna Monika Lewandowska-Sabat, Silje Furre Hansen, Trygve Roger Solberg, Olav Østerås, Bjørg Heringstad, Preben Boysen, Ingrid Olsaker

**Affiliations:** 1 Department of Basic Sciences and Aquatic Medicine, Faculty of Veterinary Medicine, Norwegian University of Life Sciences, 0033 Oslo, Norway; 2 Department of Food Safety and Infection Biology, Faculty of Veterinary Medicine, Norwegian University of Life Sciences, 0033 Oslo, Norway; 3 Geno Breeding and A.I. Association, Hamar, Norway; 4 Norwegian Cattle Health Services and TINE Extension Services, 1431 Ås, Norway; 5 Department of Animal and Aquacultural Sciences, Faculty of Biosciences, Norwegian University of Life Sciences, 1432 Ås, Norway

**Keywords:** Cattle, qPCR, Macrophages, Microrna sequencing, *Streptococcus agalactiae*, Subclinical mastitis

## Abstract

**Background:**

MicroRNAs (miRNAs) are short, non-coding RNAs that regulate gene expression at the post-transcriptional level and play a key role in the control of innate and adaptive immune responses. For a subclinical infection such as bovine streptococcal mastitis, early detection is a great challenge, and miRNA profiling could potentially assist in the diagnosis and contribute to the understanding of the pathogenicity and defense mechanisms. We have examined the miRNA repertoire and the transcript level of six key immune genes [*tumor necrosis factor alpha* (*TNFα*), *interleukin-1 beta* (*IL-1β*), *interleukin-6* (*IL-6*), *interleukin-8* (*IL-8*), *interleukin-10* (*IL-10*) and *transforming growth factor beta 1* (*TGFβ1*)] during the early phase response of bovine immature macrophages to in vitro infection with live *Streptococcus agalactiae*. Next generation sequencing of small RNA libraries from 20 cultures of blood monocyte-derived macrophages exposed to either one of two sequence types of *S. agalactiae* (ST103 or ST12) for 6 h in vitro and unchallenged controls was performed.

**Results:**

Analyzes of over 356 million high quality sequence reads, revealed differential expression of 17 and 44 miRNAs (*P* < 0.05) in macrophages infected with ST103 and ST12, respectively, versus unchallenged control cultures. We also identified the expression of 31 potentially novel bovine miRNAs. Pathway analysis of the differentially regulated miRNAs and their predicted target genes in the macrophages infected with ST12 revealed significant enrichment for inflammatory response and apoptosis, while significant enrichment for integrin and GABA signaling were found in ST103 infected macrophages. Furthermore, both bacterial strains regulated miRNAs involved in the alternative activation of macrophages. The transcript levels of *TNF-α*, *IL-1β*, *IL-6*, *IL-8* and *IL-10* were significantly up-regulated by both bacterial strains, however the expression of *TGFβ1* was significantly down-regulated only by ST12.

**Conclusions:**

Our study identified pathogen-induced differential regulation of miRNAs controlling inflammation and polarization in bovine macrophages. This implies that miRNAs have potential to serve as biomarkers for early detection of bacterial infection.

**Electronic supplementary material:**

The online version of this article (10.1186/s12864-018-4591-3) contains supplementary material, which is available to authorized users.

## Background

MicroRNAs (miRNAs) are an abundant class of non-coding, small RNA molecules (19–24 nt long), which bind to the 3’UTR of target mRNAs to repress the translation into protein or accelerate the decay of expressed transcripts (affecting 60% of all mRNA transcripts [1]). Microorganisms are known to alter the host transcriptome and proteome in several ways, affecting miRNA is one of several such mechanisms. For example, miRNA induced by *herpesviridae* and *Hepatitis C* virus appear to work as an escape mechanism by dampening the host’s immune system [2] and miRNA induced by the latter is currently being targeted for therapy [3]. Up-regulation of miRNA will mostly attenuate the immune response, but it is unclear if this should be understood as a pathogen escape mechanism or a survival mechanism on the part of the host to avoid immunopathology.

Only a few studies describe miRNA regulation in the context of bovine bacterial mastitis [4]. Modification of miRNA expression were reported in response to *Streptococcus uberis* infection of primary bovine mammary epithelial cells (BMEs) and circulating monocytes from blood and milk [5, 6]. In addition, profiling of the complete miRNA content (miRNome) of BMEs infected with *Staphylococcus aureus* and *Escherichia coli* [7], and milk exosomes from *S. aureus* infected cows [8], revealed several pathogen directed miRNAs with enriched role in immunity, infection and cellular processes. MiRNAs have also gained prominence as potential biomarkers for a range of infections and diseases, and more recent studies profiling serum miRNAs from a bovine *Mycobacterium avium subsp. paratuberculosis* infection model demonstrated high stability of circulating miRNAs [9].

Macrophages are critical effectors and regulators of inflammation serving as the first line of defense against invading pathogens. Intramammary infections will activate macrophages to produce pro-inflammatory cytokines [e.g. *tumor necrosis factor alpha* (*TNFα*), *interleukin-1 beta* (*IL-1β*), *interleukin-6* (*IL-6*) and *interleukin-8* (*IL-8*)] required to kill intracellular pathogens. However, the balance between pro- and anti-inflammatory signals [e.g. *interleukin-10* (*IL-10*) and *transforming growth factor beta 1* (*TGFβ1*)] is crucial for immune regulation of the inflammation and preventing chronic conditions. The presence of microbes together with the host microenvironment underlies the diversity of activation of macrophages [10], and in the bovine mammary gland, the status of differential macrophage activation may be pivotal for the defense and resolution of mastitis [11].

In this study, we examined the expression of selected genes and the miRNA profiles of immature macrophages (primary cells) infected in vitro with the ST103 or ST12 strain of *Streptococcus agalactiae*, a leading causative agent of subclinical bovine mastitis. ST103 is prevalent in farms with considerable environmental contamination, while ST12, a strain usually associated with colonization of pregnant women, has been found in cattle herds with no positive environmental samples [12, 13]. To the best of our knowledge, this study represents the first report of next generation sequencing used to profile the host miRNAs response to *S. agalactiae*. Furthermore, the identification of differentially expressed miRNAs in infected immature macrophages may provide a basis for development of biomarker assays for early detection of subclinical mastitis.

## Methods

### Animals and cell isolation

Six healthy Norwegian Red (NR) cows aged 4–7 years were used for miRNAs sequencing, and six healthy NR cows aged 2.5–7 years were used for reverse transcription-quantitative PCR (RT-qPCR). Five of the cows were included in both experiments (for details see Additional file 1: Table S1 and Additional file 2: Figure S1). All animals were maintained under uniform housing conditions and nutritional regimens at the Norwegian University of Life Sciences (NMBU) herd. Blood sampling was performed by certified personnel and conducted in agreement with the provisions enforced by the Norwegian Animal Research Authority. Five hundred ml of blood was collected from the neck of each animal in sterile glass bottles with sodium citrate as anticoagulant. Peripheral blood mononuclear cells (PBMC) were extracted by density gradient centrifugation (2210×g, 30 min) on lymphoprep (Axis-Shield, Norway). CD14+ cells were extracted by positive selection of monocyte differentiation antigen CD14 using anti-human CD14 MACS MicroBeads (Miltenyi Biotec GmbH, Bergisch Gladbach, Germany), according to the manufacturer’s instructions and as described earlier by Lewandowska-Sabat et al. [14]. Purity of selected cells was verified by flow cytometry by staining positively selected cells with PE conjugated anti-mouse IgG2a (Southern Biotech, Birmingham, AL, USA), analyzing in a Gallios flow cytometer (Beckman Coulter), and found to be in the range of 95–98%. The CD14+ cells were subsequently grown in 6-well dishes at a density of 1.5 × 10^6^ cells per well in RPMI medium supplemented with 10% FCS (Invitrogen, Carlsbad, USA). Cells were left over night at 37 °C in an atmosphere with 5% CO_2_. The phenotypic morphology of cells, i.e. differentiation of monocytes into an early-stage adherent macrophage phenotype, was visualized and confirmed by phase contrast microscopy.

### Bacterial infection

Two previously described *S. agalactiae* strains (ST103 and ST12) were obtained from The Norwegian Veterinary Institute [12]. These bovine adapted strains were originally isolated from milk samples. Bacteria were collected from blood agar plates and grown in “Todd Hewitt broth” (Sigma-Aldrich) until mid-log phase. Growth was measured by optical density (OD) at 600 nm. The cultures were further aliquoted and frozen in 20% glycerol stocks in − 70 °C, and the final number of colony-forming units (CFU) was determined by serial dilutions and plating on blood agar plates. Bacteria used in this study all came from aliquots of the same batch.

For each individual animal the wells with immature macrophages were grouped into four classes with as equal number of wells and cells per class as possible. Two classes were infected with ST103 or ST12, in a multiplicity of infection (MOI) of 1 (1 bacterium per cell, on average). The third cell class (positive control) was exposed to 1 mg/mL of lipopolysaccharides (LPS, rough strains) from *Salmonella minnesota* Re 595 (Re mutant, Sigma-Aldrich) and the last class was left uninfected (negative control). After 1 h of exposure, 1% of penicillin/streptomycin (60 pg/mL penicillin and 100 μg/mL streptomycin) were added to prevent growth of remaining extracellular bacteria. The controls and the infected cells were treated equally. Inhibition of bacterial growth by antibiotics was verified by microscopy. Incubation was continued for one additional hour for LPS-exposed cells and for 5 more hours for bacteria infected and negative control cell classes, a total of 6 h incubation (Additional file 2: Figure S1). Media was aspirated and the cells were collected using cell scraper. Cells were centrifuged (400×g, 5 min), the pellet was washed with cold PBS buffer, snap frozen in liquid nitrogen and stored at − 70 °C.

### RNA extraction and reverse transcription-quantitative PCR

Twenty-four RNA samples from six animals were used (Additional file 1: Table S1, Additional file 2: Figure S1). Total RNA was isolated from control and infected cells using the MirVANA isolation kit (Ambion, Austin, TX) following the manufacturer’s instructions. All RNA samples were treated with amplification grade DNase I (Invitrogen) to remove any traces of genomic DNA. RNA concentration and quality was measured using NanoDrop 1000 (Thermo Fisher Scientific, Wilmington, USA) and 2100 BioAnalyzer (Agilent RNA 6000 Nano, Agilent Technologies, Palo Alto, USA), respectively. The RNA integrity numbers (RIN), concentrations and OD A260/A280 ratios are listed in Additional file 1: Table S1. For RT-qPCR a total of 200 ng RNA was used for cDNA synthesis using Tetro cDNA synthesis kit (Nordic BioSite, Norway), and cDNA equivalent to 5 ng of total RNA was used in qPCR reactions set up in triplicate for each sample using Express SYBR GreenER SuperMix with premixed ROX (Invitrogen) according to the manufacturer’s recommendations. Transcript levels were analyzed using a 7900HT Fast Real-Time PCR System (Applied Biosystems) and the standard program: 50 °C for 2 min, 95 °C for 2 min, 40 cycles of 95 °C for 15 s and 60 °C for 1 min, followed by melting curve analyses. Gene-specific primers were either derived from literature or designed using Primer3 ver. 0.4.0 [15]. The transcript levels of the following genes were analyzed: *TNF-α*, *IL-1β*, *IL-6*, *IL-8*, *IL-10* and *TGFβ1*. The primer sequences are presented in Additional file 3: Table S2. The efficiencies of all primer pairs were tested by template dilution series using pooled cDNA from control and infected cells and were 100% (±10). Negative controls with no added template were included for all primer pairs (no template control), and no RT control reactions for each sample and each primer pair were run in qPCR in order to check for genomic DNA contamination (no RT control). *The peptidylprolyl isomerase A* (*PPIA*) housekeeping gene were used in the current study, as it has been shown to be one of the most stable genes for gene expression studies in cattle macrophages [14] and lymphocytes [16], and in human LPS-stimulated monocytes [17]. In the experiment, *PPIA* was expressed at the same level in the cells stimulated with both bacterial strains and LPS as in the negative controls. Initial analysis of the RT-qPCR data was performed using RQ Manager 1.2 (Applied Biosystems). Standard deviation of ≤0.3 per triplicate was accepted. The ΔCt method was used to calculate RT-qPCR data, i.e. ΔCt = Ct_target gene_ – Ct_reference gene_, and normalized gene expression was calculated as 2 ^(-ΔCt)^. Fold change was calculated relative to the negative, unexposed control. Reciprocal values of fold change were used for down-regulated gene expression (i.e. *TGFβ1*) in order to facilitate the interpretation of the results. The differences of normalized gene expression levels between control and infected cells for each gene were tested using Wilcoxon matched-pairs signed rank test using GraphPad Prism version 7.00 for Windows (GraphPad Software, La Jolla California USA, www.graphpad.com). The pairwise differences between responses to LPS, ST103 and ST12 were tested using log-transformed fold change values for each treatment and gene by RM one-way ANOVA and Tukey’s multiple comparison test using GraphPad Prism. The significance level was determined at *P* < 0.05.

### MicroRNA sequencing and data analyses

Twenty RNA samples from six animals were used (control = 6, LPS = 4, ST12 = 5, ST103 = 5; see Additional file 1: Table S1 and Additional file 2: Figure S1 for details) for short non-coding RNA deep sequencing. Total RNA was isolated from control and infected cells using the MirVANA isolation kit, which retains short RNA fragments, and RNA concentration and quality were measured as described above for RT-qPCR (see Additional file 1: Table S1 for details). RNA-seq libraries were prepared with NEBNext® Multiplex Small RNA Library Prep Set for Illumina (New England Biolabs), according to the manufacturer’s protocol. Libraries were sequenced (75 bp single-end, 3–400 million reads) on one lane with an Illumina NextSeq 500 machine. Preparation of RNA-seq libraries and sequencing were performed by the Norwegian Sequencing Centre (Oslo, Norway; http://www.sequencing.uio.no/).

The miRNA sequence data was analyzed using Oasis ver. 2.0, a web application that allows for fast and flexible online analysis of small-RNA-seq (sRNA-seq) data ([18]; http://oasis.dzne.de). Preliminary quality control analysis of the 20 fastq files was carried out with FASTQC software ver. 0.11.2 (http://www.bioinformatics.babraham.ac.uk/projects/fastqc/). Cutadapt ver. 1.2.1 (https://cutadapt.readthedocs.io/en/stable/) was then used to trim 3′ adaptor sequences. Reads, shorter than 17 and longer than 32 nucleotides after trimming were discarded. Preprocessed reads, which successfully passed filtering were aligned to the bovine reference genome assembly (bostau8) using STAR ver. 2.30.0e_r291 [19] in non splice-junction-aware mode, allowing 5% mismatch of the read length (0 mismatches for reads with length 17–19, 1 mismatch for reads with length 20–32). Reads that could only be aligned using soft trimming (trimming of the beginning or end of reads that is not counted as mismatches) were removed from STAR’s output using a custom script. Quantification of uniquely mapped reads was performed using featureCounts ver. 1.4.6 [20], and assignment to the short RNA species was performed using miRBase ver. 21 (release date: 2014–6-22) and Ensembl (v84).

In addition to profiling the expression of known miRNAs, the prediction of potentially novel miRNAs was performed by miRDeep2 ver. 2.0.0.5 software [21] on the combined set of all input fastq files (i.e., all the input samples were merged into a unique file containing the total reads). miRDeep2 analysis were performed with the default parameters except for the minimum read depth (−a). This parameter is automatically computed according to the total number of uniquely mapped reads, targeting a performance of at least 80% true positives and 80% recall. Further filters were applied in order to identify those novel miRNAs that have the highest likelihood of being true positives. These are based on significant randfold *p*-value (default mirdeep2 threshold), miRDeep2 score > 5, where both the mature and star read counts were expressed with a minimum of 5 reads each and rFAM alerts for other types of RNA (e.g. rRNAs and tRNAs). As Oasis maps to the mature miRNA sequence, miRDeep2 code was modified in order to show the mature miRNA position instead of the pre-mature miRNA position.

### Differential expression analysis

In order to identify statistically significant differentially expressed (DE) miRNAs between control and treatment samples, DESeq2 ver. 1.4.5. [22] was applied. The analyses were performed pairwise, i.e. negative control and one of the treatment groups at the time (LPS, ST103 or ST12, respectively) were analyzed. In addition, a pairwise comparison between the two bacterial treatment groups (i.e. ST103 and ST12) was performed. The miRNA read counts identified by miRDeep2 were normalized across all samples using DESeq2 normalization method [22]. DE miRNAs were defined as having a Benjamini and Hochberg corrected *P*-value of < 0.05 and with the minimum number of miRNAs (mean expression values) with an average of 5 reads for either biological condition. Principal component analysis (PCA) and hierarchical clustering of the samples were performed in DESeq2, in order to determine how well samples cluster together based on the similarity of their sRNA expression. The analyses were performed using Oasis.

### Target prediction and pathway analysis

DE miRNAs identified in ST103 (*n* = 17) vs. negative control and in ST12 (*n* = 44) vs. negative control were used as input lists in Ingenuity Pathway Analysis (IPA; https://www.qiagenbioinformatics.com/products/ingenuity-pathway-analysis/) in order to identify biological functions and networks that were overrepresented in the datasets.

Target genes that are potentially regulated by DE miRNAs were predicted using TargetScan ver.7.1 [23]. Given the high false positive rates for miRNA target prediction, we identified only those potential target genes that had cumulative weighted context++ score < − 0.5 [23].

Identification of statistically overrepresented biological pathways in the lists of target genes of the DE miRNAs were performed by pathway analysis using InnateDB, a curated database of innate immunity genes, pathways and molecular interactions (www.innatedb.com; [24]). Target genes predicted by TargetScan were converted to human homologs and further analyzed in InnateDB. The pathway overrepresentation analyses were performed using hypergeometric test and Benjamini and Hochberg corrected *P*-value ≤0.1 was defined as significant.

## Results

### Reverse transcription-quantitative PCR

**Fig. 1:**
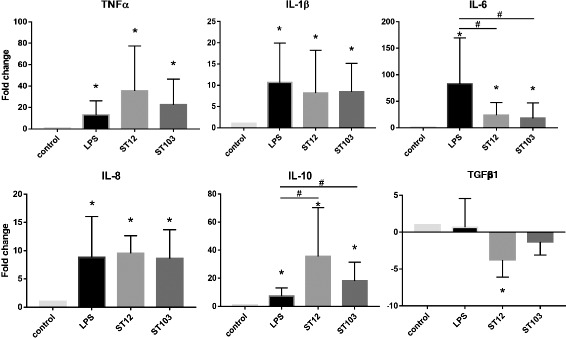
mRNA gene expression levels in bovine monocyte-derived macrophages as determined by reverse transcription-quantitative PCR (RT-qPCR). Genes displaying a significant difference in expression between the *Streptococcus agalactiae* strains ST103 and ST12, and the lipopolysaccharides (LPS) stimulated cells, respectively, compared to uninfected controls are denoted with * (*P* ≤ 0.05). The significant differences between responses to LPS vs. ST103, and LPS vs. ST12, respectively, are denoted with # (*P* ≤ 0.05). Data are presented as fold change relative to negative control, and as a mean values with SD. *Tumor necrosis factor alpha* (*TNFα*), *interleukin-1 beta* (*IL-1β*), *interleukin-6* (*IL-6*), *interleukin-8* (*IL-8*), *interleukin-10* (*IL-10*) and *transforming growth factor beta 1* (*TGFβ1*). *Peptidylprolyl isomerase A* (*PPIA*) was used as a reference gene

To assess the efficiency and quality of inflammatory responses in vitro, we measured the transcription of six major macrophage-associated cytokines by RT-qPCR, following co-incubation of macrophages with either of the two *S. agalactiae* strains ST103 or ST12, LPS or control medium. *TNF-α*, *IL-1β*, *IL-6*, *IL-8* and *IL-10* were significantly up-regulated by LPS, ST12 and ST103 compared to the controls (*P* ≤ 0.05, Fig. 1). *TGFβ1* were significantly down-regulated only in the ST12 infected macrophages (*P* ≤ 0.05, Fig. 1). *IL-6* and *IL-10* displayed significantly different responses both for LPS vs. ST103, and for LPS vs. ST12 (*P* ≤ 0.05, Fig. 1). When comparing the two *S. agalactiae* strains we did not observe significantly different expression of any of the six cytokines.

### Identification of small RNAs in bovine monocytes

Next, we conducted miRNA-seq on 20 sample libraries from the bovine monocyte-derived macrophages. Preliminary quality control analysis of the resulting 20 fastq files revealed that all libraries passed the quality criteria with a Phred score > 32. The sequencing resulted in a total of 356,741,900 high quality reads. Among them, 349,785,433 sequences (98%) remained after adaptor trimming. Approximately 43% of all reads were discarded due to length filtering after adaptor trimming. The proportion of reads uniquely aligning to different RNA biotypes demonstrated that miRNAs were the dominant class of small RNAs sequenced in our small RNA libraries. The majority (> 86%) of all reads in the 17 to 32 nucleotide fraction aligned to known miRNAs. Almost all the remaining reads mapped to small nucleolar RNAs (snoRNAs). The number of initial reads per sample, the percentage of trimmed reads and the percentage of uniquely mapped reads for each sample are presented in Additional file 4: Table S3.

**Fig. 2:**
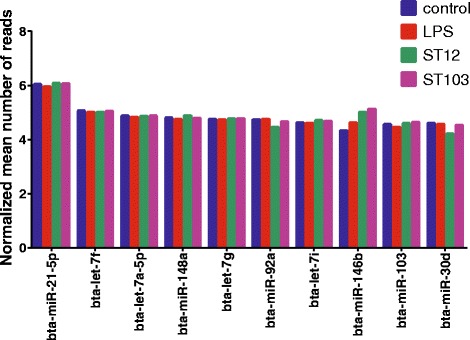
The top ten most abundant miRNAs across all samples. Bovine monocyte-derived macrophages infected in vitro with *Streptococcus agalactiae* strains ST103 or ST12 or lipopolysaccharides (LPS), and uninfected negative controls. Mean miRNAs expression values (log transformed normalized mean number of reads) are presented on the x-axis, as calculated by DESeq2 software (at least 5 mean reads for either biological condition)

The number of miRNAs with a normalized minimum mean of 5 reads detected in unchallenged macrophages were 249, while 243, 245 and 253 miRNAs were identified in LPS, ST12 and ST103 challenged macrophages, respectively (Additional file 5: Table S4). The highest expressed miRNA was bta-miR-21-5p, while four of the most abundant miRNAs belong to the bta-let-7 family (i.e., bta-let-7f, bta-let-7a-5p, bta-let-7 g and bta-let-7i). The top 10 most abundant miRNAs are presented in Fig. 2.

Applying the miRDeep2 algorithm to all 20 samples, we identified 31 high confidence, putatively novel bovine miRNAs (Table 1). Homology search in the miRBase database using the BLASTN method identified that 27 of the 31 novel miRNAs had 100% identity to known miRNAs in other species, one had a significant homology with the bta-miR-2285 family whereas three did not show significant homology to any other known miRNAs (Table 1). The high read counts observed for several of these predicted novel miRNAs suggests that the majority of them represent true novel bovine miRNAs.

**Table 1:** Novel microRNAs identified in bovine monocyte-derived macrophages with or without challenge with lipopolysaccharides (LPS), *Streptococcus agalactiae* strains ST103 and ST12

provisional ID	mature read count	star read count	miRDeep2 score	example miRBase miRNA with the same seed	consensus mature sequence	precursor coordinate
chr25_2078	63,905	8,00	32,589,40	mmu-miR-106b-3p	ccgcacuguggguacuugcugc	chr25:36892057..36892117:+
chr8_3368	48,256,00	44,00	24,641,00	hsa-let-7d-3p	cuauacgaccugcugccuuucu	chr8:86887438..86887514:+
chrX_3864	40,373,00	6,00	20,590,60	hsa-miR-223-5p	cguguauuugacaagcugaguug	chrX:99936328..99936387:-
chr7_3248	14,956,00	13,00	7636,00	mmu-miR-24-1-5p	gugccuacugagcugaaacacag	chr7:12981645..12981702:-
chr26_2162	10,315,00	54,00	5290,60	eca-miR-146b-3p	ugcccuagggacucaguucuggu	chr26:22930915..22930975:+
chr11_440	4397,00	11,00	2251,80	ssc-miR-181d-3p	accaccgaccguugacuguacc	chr11:95709449..95709509:+
chr8_3359	3633,00	95,00	1990,00	mmu-miR-27b-5p	agagcuuagcugauuggugaaca	chr8:83009841..83009902:+
chr25_2117	3826,00	7,00	1958,50	gra-miR7486h	cagcaacuaaagaucccucagg	chr25:34409297..34409357:-
chr22_1816	3325,00	78,00	1741,70	bmo-miR-3000	cugcgcuuggauuucguuccc	chr22:51543484..51543548:+
chr3_2544	1854,00	9,00	951,00	bta-miR-2285f	aaaaaccugaaugacccuuuug	chr3:94548590..94548649:+
chr8_3366	1064,00	754,00	932,60	hsa-let-7a-3p	cuauacaaucuauugccuuccc	chr8:86885231..86885309:+
chr7_3219	1392,00	74,00	748,70	hsa-miR-378a-5p	cuccugacuccagguccugugu	chr7:63067305..63067362:+
chr3_2643	863,00	137,00	513,90	hsa-miR-30c-2-3p	cugggagaggguuguuuacucc	chr3:106059376..106059437:-
chr16_1041	937,00	11,00	486,50	hsa-miR-181a-3p	accaucgaccguugauuguacc	chr16:79685955..79686018:-
chr14_712	672,00	34,00	364,10	hsa-miR-30a-3p	cuuucagucagauguuugcugcu	chr14:8080297..8080360:+
chr1_143	643,00	10,00	335,60	mmu-miR-15b-3p	cgaaucauuauuugcugcucuag	chr1:107923396..107923457:-
chr12_549	565,00	17,00	301,20	hsa-miR-92a-1-5p	agguugggaucgguugcaaugcu	chr12:66227265..66227321:+
chr1_111	475,00	6,00	249,50	mmu-miR-125b-2-3p	acaagucaggcucuugggacc	chr1:19881359..19881419:-
chr16_1003	359,00	103,00	240,00	efu-miR-9283	uguggccucuggguguguacccuc	chr16:33022297..33022356:-
chrX_3750	463,00	6,00	240,00	ssc-miR-374a-3p	uuaucagguuguauuguaauu	chrX:81951234..81951286:+
chr18_1154	297,00	42,00	181,00	hsa-let-7e-3p	cuauacggccuccuagcuuucc	chr18:58015043..58015110:+
chr4_2734	287,00	41,00	168,50	–	acacgcguccuuggauccugacu	chr4:119142851..119142912:+
chrX_3767	228,00	37,00	139,50	hsa-miR-222-5p	cucaguagccaguguagaucc	chrX:103538171..103538234:+
chrX_3861	197,00	16,00	113,20	hsa-let-7a-3p	cuauacaacuuacuacuuuccc	chrX:96382645..96382725:-
chr19_1372	157,00	5,00	82,60	–	agggagucccugguaguucagu	chr19:46741763..46741851:-
chr19_1308	103,00	22,00	65,00	–	ccccggcuuuuccucccccagg	chr19:62308493..62308542:+
chr5_2875	86,00	8,00	52,80	ssc-miR-7134-5p	auguccgcggguucccugucc	chr5:112083860..112083922:+
chr19_1281	70,00	5,00	43,00	hsa-miR-152-5p	agguucugugauacacuccgacu	chr19:39081179..39081236:+
chr18_1135	13,528,00	95,00	5,60	mmu-miR-140-5p	cagugguuuuacccuaugguag	chr18:37088153..37088219:+
chr19_1237	93,00	15,00	5,40	ahy-miR3511-5p	accagggcuggaagcugcuucu	chr19:9534051..9534107:+
chr2_1498	1928,00	20,00	5,30	hsa-miR-26b-3p	ccuguucuccauuacuuggcucg	chr2:107133408..107133466:+

### Differentially expressed miRNAs in macrophages challenged with *S. agalactiae*

The difference between the negative control and the ST103 and ST12 infected samples, respectively, was confirmed by PCA of the small RNA sequence reads. For ST103 infected samples, PC1 explained 31% and PC2 explained 22% of the overall miRNA expression variability. While for ST12 infected samples, PC1 explained 39% and PC2 explained 20% of the overall miRNA expression variability. No clear difference between the negative control and the LPS challenged samples was revealed by PCA (Additional file 6: Fig. S2).

**Fig. 3:**
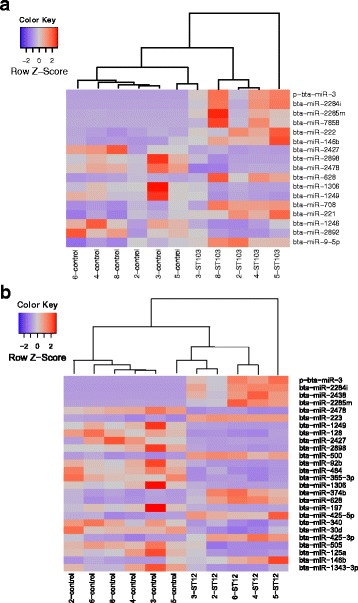
Heatmap of the top most significant differentially expressed microRNAs (*P* < 0.05) between bovine monocyte-derived macrophages infected in vitro by **a**) *Streptococcus agalactiae* strain ST103 or **b**) *Streptococcus agalactiae* strain ST12 and non-infected negative controls, as determined by DESeq2 analyses. Red color indicates high expression of the microRNA

The analyses of differential expression revealed 17 DE miRNAs in macrophages challenged with ST103 and 44 DE miRNAs in macrophages challenged with ST12, compared to the respective unchallenged negative controls (*P* < 0.05) (Table 2 and more detailed information in Additional file 7: Table S5). Moreover, 13 DE miRNAs were detected when macrophages challenged with ST12 were compared to macrophages challenged with ST103 (P < 0.05) (Additional file 7: Table S5). No significantly differently regulated miRNAs were identified in LPS challenged macrophages compared to the unchallenged controls. Heatmap analysis of the top most significant DE miRNAs in the ST103 and the ST12 challenged samples, respectively, shows that the infected samples and most of the control samples cluster into different groups (Fig. 3).

**Table 2:** MicroRNAs significantly differentially expressed between bovine monocyte-derived macrophages infected with *Streptococcus agalactiae* strains ST103 or ST12 and the respective uninfected controls

ST103			ST12		
miRNA ID	Log2FC	*P*-value	miRNA ID	Log2FC	*P*-value
p-bta-miR-3	8,22	8,77E-45	p-bta-miR-3	9,98	1,10E-53
bta-miR-2284i	7,37	2,28E-29	bta-miR-2284i	8,62	9,53E-41
bta-miR-2285m	4,97	5,87E-13	bta-miR-2438	7,93	2,46E-25
bta-miR-7858	4,24	1,15E-06	bta-miR-2285 m	6,08	9,41E-17
bta-miR-222	1,75	1,05E-05	bta-miR-2478	−2,29	4,69E-15
bta-miR-146b	2,34	1,88E-04	bta-miR-223	1,27	3,63E-14
bta-miR-2427	−2,53	1,60E-03	bta-miR-1249	−3,29	1,77E-13
bta-miR-2898	−1,52	2,88E-03	bta-miR-128	−1,91	2,34E-11
bta-miR-2478	−0,93	4,00E-03	bta-miR-2427	−4,19	1,55E-09
bta-miR-628	1,20	6,59E-03	bta-miR-2898	−2,92	2,98E-09
bta-miR−1306	−1,73	8,43E-03	bta-miR-500	1,39	4,76E-09
bta-miR-1249	−1,54	1,14E-02	bta-miR-92b	−2,27	2,08E-08
bta-miR-708	1,08	1,85E-02	bta-miR-484	−1,81	3,88E-08
bta-miR-221	0,69	3,71E-02	bta-miR-365-3p	−2,36	4,13E-07
bta-miR-1246	−0,94	4,33E-02	bta-miR-1306	−3,34	8,34E-07
bta-miR-2892	− 1,57	4,97E-02	bta-miR-374b	1,57	6,66E-06
bta-miR-9-5p	1,13	4,97E-02	bta-miR-628	1,50	7,96E-06
			bta-miR-197	−2,21	9,36E-06
			bta-miR-425-5p	0,93	3,38E-05
			bta-miR-340	−1,39	1,28E-04
			bta-miR-30d	−1,31	1,72E-04
			bta-miR-425-3p	1,04	1,82E-04
			bta-miR-505	−1,15	1,19E-03
			bta-miR-125a	−2,01	1,22E-03
			bta-miR-146b	2,11	1,64E-03
			bta-miR-1343-3p	−1,59	1,64E-03
			bta-miR-2388-5p	−2,67	2,77E-03
			bta-miR-423-3p	−0,98	2,85E-03
			bta-miR-328	−1,84	3,39E- 03
			bta-miR-30b-5p	1,26	3,63E-03
			bta-miR-92a	−0,91	6,29E-03
			bta-miR-1468	−0,88	6,32E-03
			bta-miR-30f	−1,34	6,58E-03
			bta-miR-125b	−1,18	8,86E-03
			bta-miR-10a	−0,76	9,31E-03
			bta-miR-2431-3p	−2,25	9,76E-03
			bta-miR-155	1,33	1,76E- 02
			bta-miR-361	−0,97	1,97E-02
			bta-miR-122	2,12	2,12E-02
			bta-miR-221	0,65	2,16E-02
			bta-miR-2284ab	−1,06	3,02E-02
			bta-miR-2284w	−0,74	3,02E-02
			bta-miR-30c	−0,82	3,62E-02
			bta-miR-669	−0,85	4,39E-02

Log2FC – log2 fold change values compared to controls, *P*-value - Benjamini and Hochberg corrected *P*-value

**Fig. 4:**
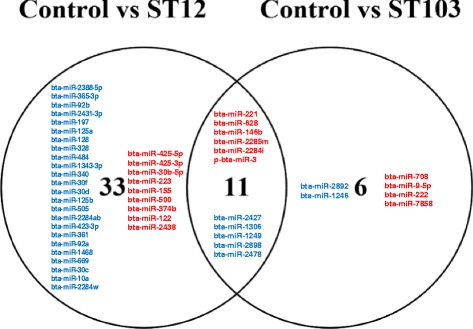
Venn diagram of differentially expressed miRNAs (*P* < 0.05) between *Streptococcus agalactiae* strain ST103 or ST12 infected and respective non-infected (negative controls) bovine monocyte-derived macrophages. Red indicates miRNAs that were up-regulated, and blue indicates miRNAs that were down-regulated compared to the negative control samples

Eleven DE miRNAs were commonly regulated by both *S. agalactiae* strains (6 up-regulated and 5 down-regulated), while 33 DE miRNAs were only regulated by ST12 (9 up-regulated and 24 down-regulated) and 6 were only regulated by ST103 (4 up-regulated and 2 down-regulated; Fig. 4). Ten out of 13 DE miRNAs between ST12 and ST103 were also found either among DE miRNAs in macrophages challenged with ST12 or with ST103, compared to the respective unchallenged negative controls. Moreover, three out of these (i.e. bta-miR-2478, bta-miR-1249 and bta-miR-2898; Additional file 7: Table S5) were found among DE miRNAs commonly regulated by both *S. agalactiae* strains.

One novel miRNA (p-bta-miR-3) was found to be DE in response to both bacterial strains (Fig. 4, Additional file 7: Table S5). This 20 nt long miRNA was mapped to bovine chromosome 9 and had a significant homology to mmu-mir-7025-5p (E-value 6.2).

### Enriched pathways of differentially regulated miRNAs and their predicted target genes

**Fig. 5:**
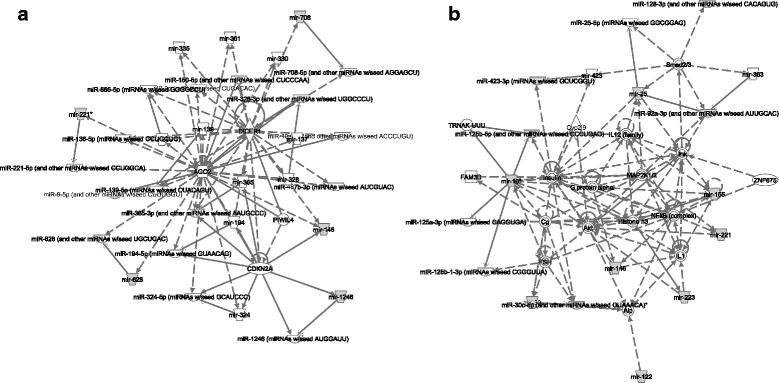
Functional network overrepresented in the list of differentially expressed miRNAs in response to infection of bovine macrophages with *Streptococcus agalactiae* strain (**a**) ST103 and (**b**) ST12. Ingenuity Pathway Analysis (IPA) identified 6 associated molecules with a network score of 16 for ST103 and 13 associated molecules with a network score of 31 for ST12

IPA was used to analyze the miRNAs that were differentially regulated in response to ST103 infection (*n* = 17) in order to identify biological networks and functions enriched in the dataset. Nine bovine miRNAs were mapped to their human miRNA homologs. The analyses revealed that the most significant biological network in the dataset with 6 focus molecules, were associated with cancer, organismal injury and abnormalities and reproductive system disease (Fig. 5a, Additional file 8: Table S6). MiRNAs differentially regulated in response to ST12 infection (*n* = 44) were also analyzed. Twenty-nine bovine miRNAs were mapped to their human miRNA homologs by IPA. The analyses revealed that the most significant biological network with 13 focus molecules were associated with connective tissue disorders, inflammatory disease and inflammatory response (Fig. 5b, Additional file 8: Table S6). Due to difficulties in accurately predicting miRNA targets, it is more common to examine the statistically overrepresented functional categories among predicted target genes than focusing on individual gene predictions. In order to identify significantly enriched pathways among the target genes of the DE miRNAs, we analyzed the up- and down-regulated target genes separately. Hence, 239 and 182 target genes of the down- and up-regulated DE miRNAs, respectively, in response to ST103, and 1003 and 463 target genes of down- and up-regulated DE miRNAs, respectively, in response to ST12 were analyzed using InnateDB. The top significantly enriched pathways are presented in Table 3. Target genes and pathways for each list of DE miRNAs are presented in Additional file 9: Table S7.

**Table 3:** Top significant pathways overrepresented among target genes of differentially expressed miRNAs in response to exposure of bovine macrophages in vitro to *Streptococcus agalactiae* strains ST103 or ST12

		Pathway name	*P*-value	*P*-value (B-H)	Genes
ST103	miRNAs up-regulated	Mucin type O-Glycan biosynthesis	1.4E-4	0.01	GALNT3; GALNT4; GALNT9; POC1B-GALNT4;
		GABA receptor activation	1.0E-4	0.02	GABRA1; GABRB2; GNG10; GNG5; KCNJ2;
		Regulation of gene expression in early pancreatic precursor cells	0.001	0.06	ONECUT1; ONECUT3;
	miRNAs down-regulated	Integrin signaling pathway	0.004	0.10	ACTN1; ACTN2; BCR; CAV1; FYN; ITGA1; MAPK8; PXN; SOS1; TNS1; VCL;
		Sodium/Calcium exchangers	0.003	0.11	SLC24A1; SLC24A2; SLC24A3; SLC24A4; SLC8A1; SLC8A2; SLC8A3;
		Reduction of cytosolic Ca++ levels	0.004	0.11	ATP2A2; ATP2B1; ATP2B2; ATP2B3; ATP2B4; SLC8A1; SLC8A2; SLC8A3;
ST12	miRNAs up-regulated	Activation of G protein gated Potassium channels	2.3E-4	0.04	GNB3; GNG10; GNG2; GNG5; KCNJ6;
		G protein gated Potassium channels	2.3E-4	0.04	GNB3; GNG10; GNG2; GNG5; KCNJ6;
		Inhibition of voltage gated Ca2+ channels via Gbeta/gamma subunits	2.3E-4	0.04	GNB3; GNG10; GNG2; GNG5; KCNJ6;
	miRNAs down-regulated	IL4-mediated signaling events	5.1E-4	0.15	BCL2L1; CCL11; CCL26; FCER2; IL10; IRF4; IRS2; SOCS1; SOCS5; STAT6;
		Intrinsic Pathway for Apoptosis	3.6E-4	0.16	BAK1; BAX; BCL2L1; BMF; CASP7; TFDP1; YWHAB; YWHAG;
		Role of parkin in ubiquitin-proteasomal pathway	3.1E-4	0.28	PARK2; UBE2E2; UBE2G1; UBE2L3;

GALNT3/4/9 - polypeptide N-acetylgalactosaminyltransferase 3/4/9; POC1B - POC1 centriolar protein B; GABRA1/B2 - gamma-aminobutyric acid type A receptor alpha1 subunit/beta2 subunit; GNG 2/5/10 - G protein subunit gamma 10/5; KCNJ2/6 - potassium voltage-gated channel subfamily J member 2/6; ONECUT1/3 - one cut homeobox 1/3; ACTN1/2: actinin alpha 1/2; BCR - RhoGEF and GTPase activating protein; CAV1 - caveolin 1; FYN - FYN proto-oncogene, Src family tyrosine kinase; ITGA1 - integrin subunit alpha 1; MAPK8 - mitogen-activated protein kinase 8; PXN - paxillin; SOS1 - SOS Ras/Rac guanine nucleotide exchange factor 1; TNS1 - tensin 1; VCL - vinculin; SLC24A1/2/3/4 - solute carrier family 24 member 1/2/3/4; SLC8A1/2/3 - solute carrier family 8 member 1/2/3; ATP2A2 - ATPase sarcoplasmic/endoplasmic reticulum Ca2+ transporting 2; ATP2B1/2/3/4 -ATPase plasma membrane Ca2+ transporting 1/2/3/4; GNB3 - G protein subunit beta 3; BCL2L1- BCL2 like 1; CCL11/26 - chemokine (C-C motif) ligand 11/26; FCER2 - Fc fragment of IgE receptor II; IL10 - interleukin 10; IRF4 - interferon regulatory factor 4; IRD2 - insulin receptor substrate 2; SOCS1/5 - suppressor of cytokine signaling 1; STAT6 - signal transducer and activator of transcription 6; BAK1 - BCL-2 antagonist killer 1; BAX - BCL-2 associated X; BMF - Bcl2 modifying factor; CASP7 - caspase 7; TFDP1 - transcription factor Dp-1; YWHAB/G - tyrosine 3-monooxygenase/tryptophan 5-monooxygenase activation protein beta/gamma; PARK2 - parkin RBR E3 ubiquitin protein ligase; UBE2E2/G1/L3 - ubiquitin-conjugating enzyme E2E2/E2G1/E2L3

## Discussion

MiRNAs are suggested to be fine-tuners of gene expression during inflammatory response [25, 26]. This indicates a great potential of the genome-wide miRNome profiling for investigation of inflammation. In this study, we have identified the early phase miRNome of bovine monocyte-derived macrophages infected in vitro with two strains of *S. agalactiae* derived from Norwegian dairy herds. *S. agalactiae* is a major causative agent of subclinical mastitis and an increasing problem in Norway. Furthermore, *S. agalactiae* is widely recognized as a main cause of life-threatening infections in human neonates, pregnant females, and elderly adults [27]. The two strains used here differ in their ability to survive in the environment and transmit within dairy herds. ST103 is the most predominant isolate in bovine mastitis found in farms with substantial environmental contamination, while ST12 was found in cattle herds with no positive environmental samples [12]. Using modern sequencing of miRNAs from macrophages infected with *S. agalactiae* and analyzing over 356 million reads, we found that several miRNAs were significantly differentially regulated in response to infection (Figs. 3 and 4). We observed that fewer miRNAs were differentially regulated in macrophages challenged with strain ST103 than with strain ST12 (17 and 44, respectively; Fig. 4). This suggests that ST103 induces relatively subtle changes in miRNA expression during the early stage of infection. However, the expression pattern of the examined pro- and anti-inflammatory genes was not significantly different between these two strains, except for *TGFβ1*, that was significantly down-regulated only by ST12 (Fig. 1).

It has been shown that different bacterial strains are often associated with a variety of virulence factors, which, in turn, are associated with the outcome of infection in the host [28, 29]. The latest study of molecular and virulence characteristics of the most prevalent bovine *S. agalactiae* isolates has shown that these factors in strain ST103 (but also in other of the most prevalent bovine isolates) are associated with growth ability in milk, biofilm formation and adhesion to bovine mammary epithelial cells. Furthermore, ST103 showed significantly higher hemolytic activity and cytotoxicity compared to most of the other bovine strains [30]. On the contrary, the characterization of ST12 revealed that this strain shows high rates of resistance to erythromycin and clindamycin [31] and can survive within macrophages [32]. The observed differences in the miRNA expression profiles during the early stage of macrophage infection may be explained by the different characteristics of these two *S. agalactiae* strains such as the differences in virulence factors, the different niches and modes of transmission.

Both strains increased the level of miR-146b and IL-10 transcripts. This may be associated with toll-like receptor (TLR) recognition of bacterial patterns, as shown in monocytes for other bacterial TLR-agonists. MiR-146b has been reported to reduce the expression of several pro-inflammatory cytokines and chemokines in human monocytes upon stimulation with LPS [33], which suggests that miR-146b has an anti-inflammatory activity in monocytes. In a previous transcriptomic study, we found indications that another gram-positive bacterial mastitis pathogen, *S. aureus*, promotes bovine macrophages to be activated in an alternative manner [14]. Here we observed several indications of alternative activation on the miRNA level as a result of streptococcal uptake in macrophages. Both strains induced miR-221, ST12 induced miR-30b, miR-223, miR-374b and miR-500 but down-regulated miR-125a and miR-125b, while ST103 induced miR-222, all associated with alternative macrophage activation [34–37]. While one study reported that miR-125a promotes classical macrophage activation [38], and several of the DE miRNA found in our study are not accounted for in the literature, the results suggest that *S. agalactiae* may induce an alternative-like macrophage activation similar to *S. aureus*. However, this result needs to be confirmed by more comprehensive functional studies.

One of the novel miRNA (p-bta-miR-3) was found to be DE in response to both bacterial strains (Fig. 4). This miRNA shares 100% sequence identity and covers 95% of the query (i.e. 19 out of 20 nucleotides match) when blasted against the genome sequences of several *S. agalactiae* strains (e.g. GenBank: CP025028.1; https://blast.ncbi.nlm.nih.gov/Blast.cgi). This suggests that it may be a bacterial-derived miRNA released by infection of the macrophages. Bacterial-derived miRNAs have recently been extensively studied [39, 40]. It is likely that these small RNA molecules may repress the ability of the host cell to resist bacterial invasion, as has been shown for viruses and fungi [41–43]. It is not clear if bacterial miRNAs require the host cell miRNA processing machinery for its biogenesis or if it is a bacterial RNA fragment released into the host cell cytoplasm during bacterial lysis. Whether p-bta-miR-3 observed in our study is an authentic miRNA of bacterial origin needs to be further tested.

Mapping of bovine miRNAs to their human homologs may reveal conserved pathways and biological functions associated with the regulation of gene expression during immune response to bacteria. To characterize the distinct DE miRNA profiles of the two bacterial strains we performed pathway analysis using IPA. As a result 2 genes, *dicer 1 ribonuclease III* (*DICER1*) and *argonaute 2* (*AGO2*), were found to be associated with the ST103 DE miRNAs (e.g., miR-9-5p and miR-708; Fig. 5a). *DICER* and *AGO2* are miRNA processing enzymes, and *DICER1* knockdown cell lines and animals have been correlated with a global drop of mature miRNA expression levels, shifting macrophages towards a classically activated phenotype [44–46]. These results show that strain ST103 induces miRNAs that likely contribute to maintain an alternative macrophage phenotype.

For miRNAs down-regulated in ST103 infected macrophages, putative target genes with a role in the integrin signaling pathway may be noted (Table 3). Integrin activation is necessary for leukocyte arrest before migration across the endothelial cell barrier and many bacterial species, such as group A streptococci [47] *S. aureus* [48], and *E. coli* [49] use host integrins for adhering to and invading host cells. Moreover, the study of virulence factors of bovine *S. agalactiae* isolates has identified virulence factors associated with adhesion ability to bovine mammary epithelial cells in strain ST103 [30]. These findings, together with our results may indicate that the integrin signaling pathway is essential for *S. agalactiae* ST103 strain invasion of macrophages or mammary epithelial cells.

Furthermore, target genes of ST103 up-regulated miRNAs are involved in gamma-aminobutyric acid (GABA) receptor activation (Table 3). GABA plays an inhibitory role in autoimmune inflammation [50] and *GABA* receptor transcripts are present in different immune cells [50–53]. Inflammatory cytokine production in peripheral macrophages and T cell autoimmunity is decreased after GABA treatment [51, 52]. Moreover, GABA and GABA type A receptor agonists reduced cytotoxic immune responses of T cells [54]. Other target genes associated with up-regulated ST103 miRNAs belong to cancer, organismal injury, abnormalities, and reproductive system disease pathways, without obvious connection to the bacterial infection context (Additional file 8: Table S6). Taken together, ST103 induced miRNA regulation of integrin and GABA signaling, and maintenance of alternative macrophage activation suggest that miRNAs may be involved in the pathogenesis of subclinical mastitis. However, whether this is an evasion strategy developed by *S. agalactiae* ST103 to avoid host defense and promote its intracellular replication and persistence remains to be determined.

In contrast, IPA revealed that strain ST12 induced DE miRNAs were associated with inflammatory disease and inflammatory response (Fig. 5b, Additional file 8: Table S6). A number of genes associated with the ST12 induced miRNA networks are key players of immune response, i.e. *IL-1*, *nuclear factor kappa β* (*NFKβ*), *interleukin-12* (*IL-12*), *Janus kinase* (*JaK*) and *SMAD family member 2/3* (*Smad2/3*) (Fig. 5b). For three of the ST12-unique DE miRNAs (bta-miR-155, bta-miR-125b and bta-miR-223) evidence of induction by bacteria has been previously documented. It has been shown that miR-155 and miR-125b are involved in regulation of *TNF* production during mycobacterial infection [55, 56]. MiR-155 was reported to induce toll-like receptor 2 (TLR-2) recognition of *Streptococcus pneumoniae*, and promote effective bacterial clearance in the nasopharynx [57]. MiR-223 regulates inflammation (reviewed in [58]) and was found to be up-regulated in bovine mammary tissue infected with *S. uberis* [59]. Moreover, an earlier study shows that *Salmonella* can up-regulate intestinal epithelial miR-128 expression, which, in turn, decreases macrophage recruitment [60]. Finally, IPA revealed that miR-128 and miR-92a are associated with Smad2/3 proteins (Fig. 5b), critical downstream mediators of TGFβ-signaling (reviewed in [61]). This is consistent with the qPCR findings of down-regulation of *TGFβ1* by ST12 (Fig. 1).

The predicted target genes of the down-regulated miRNAs in ST12 infected macrophages were significantly enriched for genes with roles in interleukin-4 (IL-4)-mediated signaling events and intrinsic pathway for apoptosis (Table 3). These genes have multiple roles in regulation of apoptosis [e.g. *BCL-2 like 1* (*BCL2L1*), *BCL-2 antagonist killer 1* (*BAK1*), *BCL-2 associated X* (*BAX*)] and anti-inflammation (e.g. *IL-10*), and display chemotactic activity for eosinophils [e.g. *C-C motif chemokine ligand 11* (*CCL11*), *C-C motif chemokine ligand 26* (*CCL26*)]. Pro-apoptotic *BAK1* is a direct target of miR-125b [62] and in our study miR-125b was found to be down-regulated in response to ST12 (Fig. 4). Apoptosis plays a critical role in the pathogenesis of sepsis [63] and *S. agalactiae* strains are frequent agents of life-threatening sepsis and meningitis in human neonates and adults. This may indicate the putative mechanisms of pathogenesis, i.e. impaired immune responses due to extensive death of immune system cells [63]. However, further in vivo studies are needed in order to confirm this hypothesis.

The predicted target genes of ST12 up-regulated miRNAs have multiple roles in G protein signaling (Table 3). It has been shown that G-protein-coupled receptors (GPCRs) are linked to heterotrimeric G-proteins composed of α, β, and γ subunits (reviewed in [64]). Inflammatory cells such as macrophages express a large number of GPCRs for classic chemoattractants and chemokines, and these receptors are critical for both enhancement of inflammation and promotion of its resolution (reviewed in [65]). This may suggests that ST12-mediated up-regulation of miRNAs, which in turn regulate G-protein genes, may have impact on regulation of inflammatory gene expression, particularly those facilitating chemotaxis. Taken together, we found that strain ST12 induces a stronger inflammatory immune response in bovine macrophages than strain ST103, with miRNAs associated with down-regulation of the TGFβ/Smad signaling, chemotactic pathways and enhancement of apoptotic pathways and alternative macrophage activation.

Three out of thirteen DE miRNAs (i.e. bta-miR-2478, bta-miR-2898 and bta-miR-1249) detected among macrophages challenged with ST12 compared to macrophages challenged with ST103 were also found among DE miRNAs commonly regulated by both *S. agalactiae* strains (Fig. 4 and Additional file 7: Table S5). It was reported recently that bta-miR-2478 inhibits *TGFβ1* expression during mammary gland development in goats and a strong negative correlation between miR-2478 and *TGFβ1* expression was demonstrated [66]. We could not observe this negative correlation in our study, as the lowest transcript level of *TGFβ1* (Fig. 1) was associated with the lowest expression of bta-miR-2478 (Additional file 5: Table S4) in macrophages challenged with ST12, compared to the other groups. However, it has also been reported that this miRNA is associated with energy metabolism and feeding-induced changes in muscles, and regulates different target genes in cattle [67, 68]. This suggests that bta-miR-2478 may target other gene(s) than *TGFβ1* in infected macrophages. Bta-miR-2898 was found to be up-regulated in the mammary gland tissues of mastitis-infected cows, while bta-miR-1249 was identified as miRNA, which targets genome of H5N1 influenza A virus during in vitro infection [69, 70]. The significantly stronger down-regulation of these three miRNAs in macrophages challenged with ST12 compared to ST103 may be explained partly by the differences in virulence factors between these strains and suggest an important role of these miRNAs during the early stage of macrophage infection.

We were not able to identify any significant DE miRNAs in response to LPS in our study, however, several pro- and anti-inflammatory genes were up-regulated after exposing macrophages to LPS (Fig. 1). This may be due to higher individual variation in miRNA expression between LPS-treated samples combined with lower number of biological replicates (*n* = 4) compared to ST12 and ST103 (Additional file 6: Figure S2). However, we were not able to identify any outliers within the LPS-treated group based on the variation pattern (Additional file 6: Figure S2), in order to improve the statistical analysis. The number of biological replicates needed to ensure valid biological interpretation of high-throughput sequencing results is unclear. However, some studies attempt to answer this question and provide guidelines for experimental design. Earlier it has been proposed that the absolute minimum of three biological replicates should be used for next-generation sequencing studies, however, Schurch et al. [71] suggested that at least six biological replicates should be used in order to assure statistical power in the detection of differentially expressed genes. In the present study, we used four replicates for LPS-stimulated and five replicates for each of the bacterial strain-challenged macrophages. Furthermore, using five biological replicates for ST103- and ST12-treated samples may also have a negative impact on the number of detected significant miRNAs. Increasing the number of biological replicates would be beneficial to increase the statistical power and accuracy of the RNA and miRNA-seq analyses, but unfortunately, this was not possible within the scope of the present project. Originally, we had planned for six samples of each class of exposure, but some of the cultures failed to yield sufficient number of cells.

We observed approximately 250 miRNAs expressed in bovine macrophages (Additional file 5: Table S4). This is in agreement with the previous reports on miRNA sequencing of primary bovine mammary epithelial cells infected with *S. uberis* [6] and with *S. aureus* and *E. coli* [7], but it is approximately 3 times more than identified in bovine alveolar macrophages [72]. In our study, some miRNAs were expressed at very high levels (Fig. 2), while the majority were expressed at low levels. This is also observed in several previous studies of macrophages and epithelial cells miRNome [6–8, 72].

## Conclusions

Our study have shown that *S. agalactiae* strain ST12 induces miRNAs associated with a stronger inflammatory response than *S. agalactiae* strain ST103. Both seem to drive the macrophages away from a classical (M1) type of activation, but further studies are needed to classify them into canonical activation phenotypes. Taken together these analyses suggest that the differentially expressed miRNAs identified in this study during in vitro infection of bovine macrophages with two different strains of *S. agalactiae* likely are crucial regulators of the innate immune response to this pathogen, and thus represent potential biomarkers of infection and inflammation. We have also identified several candidate pathways likely involved in the pathogenesis of subclinical mastitis. The study contributes to better understanding of the pathogenic mechanisms of different bacteria strains within one species.

## Additional files

Additional file 1: **Table S1.** RNA samples that were used in the study. RIN - RNA integrity number; RT-qPCR - reverse transcription-quantitative PCR; miRNAs seq - microRNA sequencing. X indicates which samples were used in each of the experiments. (DOCX 16 kb)

Additional file 2: **Figure S1.** Experimental design of reverse transcription-quantitative PCR (RT-qPCR) and microRNA sequencing experiments. S. agal: samples of in vitro exposure of blood monocyte-derived macrophages with live *Streptococcus agalactiae* strain ST103 or strain ST12, respectively; Neg.con: negative control: sample of uninfected blood monocyte-derived macrophages; 6 h – 6 h infection with *Streptococcus agalactiae*, 2 h – 2 h exposure to LPS. For details on the experimental design, see the Materials and Methods section. (PDF 134 kb)

Additional file 3: **Table S2.** List of primers used for reverse transcription-quantitative PCR (RT-qPCR). (DOCX 14 kb)

Additional file 4: **Table S3.** The results of next generation sequencing of 20 libraries from bovine monocyte-derived macrophages. The number of initial reads per sample, the percentage of trimmed reads, the percentage of uniquely mapped reads, read distribution (% reads), length filtering (% of discarded reads) and an average read length (nt) for each sample are presented. (XLSX 14 kb)

Additional File 5: **Table S4.** The lists of microRNAs identified in blood monocyte-derived macrophages infected in vitro with live *Streptococcus agalactiae* strains ST103 and ST12, LPS, and uninfected (controls), respectively. Mean reads number normalized across all samples were calculated using DESeq2. (XLSX 71 kb)

Additional file 6: **Figure S2.** Principal component analysis of mapped sequence reads. Control represents sequences from non-infected bovine monocyte-derived macrophage libraries, and a) *Streptococcus agalactiae* strain ST103 infected; b) *Streptococcus agalactiae* strain ST12 infected; c) LPS-challenged; and d) *Streptococcus agalactiae* strain ST103 or strain ST12 infected macrophages isolated from the same animals. (PDF 264 kb)

Additional file 7: **Table S5.** Detailed information on microRNAs significantly differentially expressed between bovine monocyte-derived macrophages infected with *Streptococcus agalactiae* strains ST103 or ST12, and the respective uninfected controls. Padj - Benjamini and Hochberg corrected *P*-value. (XLSX 17 kb)

Additional file 8: **Table S6.** Biological networks associated with top diseases and functions that were overrepresented among microRNAs differentially expressed between bovine monocyte-derived macrophages infected with *Streptococcus agalactiae* strains ST103 or ST12, and the respective uninfected controls. (XLSX 10 kb)

Additional file 9: **Table S7.** List of target genes of the differentially expressed miRNA as predicted by TargetScan (cumulative weighted context++ score < − 0.5) in ST103 or ST12 infected bovine macrophages and the list of overrepresented pathways among these target genes as analyzed by InnateDB. (XLSX 410 kb)

## Abbreviations

AGO2: Argonaute 2
BAK1: BCL-2 antagonist killer 1
BAX: BCL-2 associated X
BCL2L1: BCL-2 like 1
BMEs: Bovine mammary epithelial cells
CCL11: C-C motif chemokine ligand 11
CCL26: C-C motif chemokine ligand 26
CFU: Colony-forming units
DE: Differentially expressed
DICER1: Dicer 1, ribonuclease III
GABA: Gamma-aminobutyric acid
GPCRs: G-protein-coupled receptors
IL-10: Interleukin-10
IL-12: Interleukin-12
IL-1β: Interleukin-1 beta
IL-4: Interleukin-4
IL-6: Interleukin-6
IL-8: Interleukin-8
IPA: Ingenuity Pathway Analysis
JaK: Janus kinase
LPS: Lipopolysaccharides
M1: Classically activated macrophages
miRNA: microRNA
miRNome: Complete miRNA content
MOI: Multiplicity of infection
NFKβ: Nuclear factor kappa β
NMBU: Norwegian University of Life Sciences
NR: Norwegian Red
OD: Optical density
PBMC: Peripheral blood mononuclear cells
PCA: Principal component analysis
PPIA: Peptidylprolyl isomerase A
RIN: RNA integrity numbers
RT-qPCR: Reverse transcription-quantitative PCR
SMAD2/3: SMAD family member 2/3
small nucleolar RNAs: snoRNAs
sRNA-seq: Small-RNA-seq
TGFβ1: Transforming growth factor beta 1
TLR: Toll-like receptor
TLR2: Toll-like receptor 2
TNFα: Tumor necrosis factor alpha
