# Supplementary material for: MicroRNA expression profiles of bovine monocyte-derived macrophages infected in vitro with two strains of Streptococcus agalactiae
Source: BMC Genomics. 2018 Apr 10;19:241. doi: 10.1186/s12864-018-4591-3 (PMC5894239; doi:10.1186/s12864-018-4591-3)
Supplement: Supplementary file 1 — Table S1. RNA samples that were used in the study. RIN - RNA integrity number; RT-qPCR - reverse transcription-quantitative PCR; miRNAs seq - microRNA sequencing. X indicates which samples were used in each of the experiments. (DOCX 16 kb) [file 12864_2018_4591_MOESM1_ESM.docx]

| Sample Name | Conc. (ng/µl) | A260/280 | RIN | RT-qPCR | miRNA seq |
| --- | --- | --- | --- | --- | --- |
| 2_control | 110.9 | 2.0 | 8.5 | x | x |
| 2_LPS | 88.7 | 1.8 | 8.6 | x | x |
| 2_ST12 | 84.2 | 2.0 | 7.6 | x | x |
| 2_ST103 | 72.2 | 1.8 | 8.7 | x | x |
| 3_control | 107.2 | 2.0 | 7.8 | x | x |
| 3_LPS | 93.4 | 2.0 | 7.5 | x | x |
| 3_ST12 | 95.7 | 2.0 | 7.0 | x | x |
| 3_ST103 | 68.5 | 1.9 | 6.6 | x | x |
| 4_control | 78.1 | 2.4 | 6.8 | x | x |
| 4_LPS | 37.9 | 3.4 | 6.3 | x |  |
| 4_ST12 | 118.7 | 2.4 | 7.8 | x | x |
| 4_ST103 | 77.9 | 2.4 | 6.8 | x | x |
| 5_control | 72.4 | 2.5 | 8.1 | x | x |
| 5_LPS | 52.9 | 3.8 | 7.9 | x | x |
| 5_ST12 | 164.2 | 2.1 | 7.1 | x | x |
| 5_ST103 | 87.9 | 2.5 | 8.2 | x | x |
| 6_control | 163.7 | 2.1 | 7.3 |  | x |
| 6_ST12 | 81.3 | 1.9 | 9.1 |  | x |
| 8_control | 57.8 | 2.0 | 7.7 | x | x |
| 8_LPS | 83.6 | 2.0 | 6.7 | x | x |
| 8_ST12 | 53.6 | 2.0 | N/A | x |  |
| 8_ST103 | 124.5 | 1.9 | 7.1 | x | x |
| 9_control | 110.1 | 2.0 | 8.2 | x |  |
| 9_LPS | 89.6 | 2.1 | 7.4 | x |  |
| 9_ST12 | 86.6 | 2.0 | N/A | x |  |
| 9_ST103 | 57.3 | 2.1 | 7.3 | x |  |

**Table S1.**

RNA samples that were used in the study. RIN - RNA integrity number; RT-qPCR - reverse transcription-quantitative PCR; miRNA seq - microRNA sequencing. X indicates which samples have been used in each of the experiments.
