# Supplementary material for: MicroRNA expression profiles of bovine monocyte-derived macrophages infected in vitro with two strains of Streptococcus agalactiae
Source: BMC Genomics. 2018 Apr 10;19:241. doi: 10.1186/s12864-018-4591-3 (PMC5894239; doi:10.1186/s12864-018-4591-3)
Supplement: Supplementary file 2 — Figure S1. Experimental design of reverse transcription-quantitative PCR (RT-qPCR) and microRNA sequencing experiments. S. agal: samples of in vitro exposure of blood monocyte-derived macrophages with live Streptococcus agalactiae strain ST103 or strain ST12, respectively; Neg.con: negative control: sample of uninfected blood monocyte-derived macrophages; 6 h – 6 h infection with Streptococcus agalactiae, 2 h – 2 h exposure to LPS. For details on the experimental design, see the Materials and Methods section. (PDF 134 kb) [file 12864_2018_4591_MOESM2_ESM.pdf]

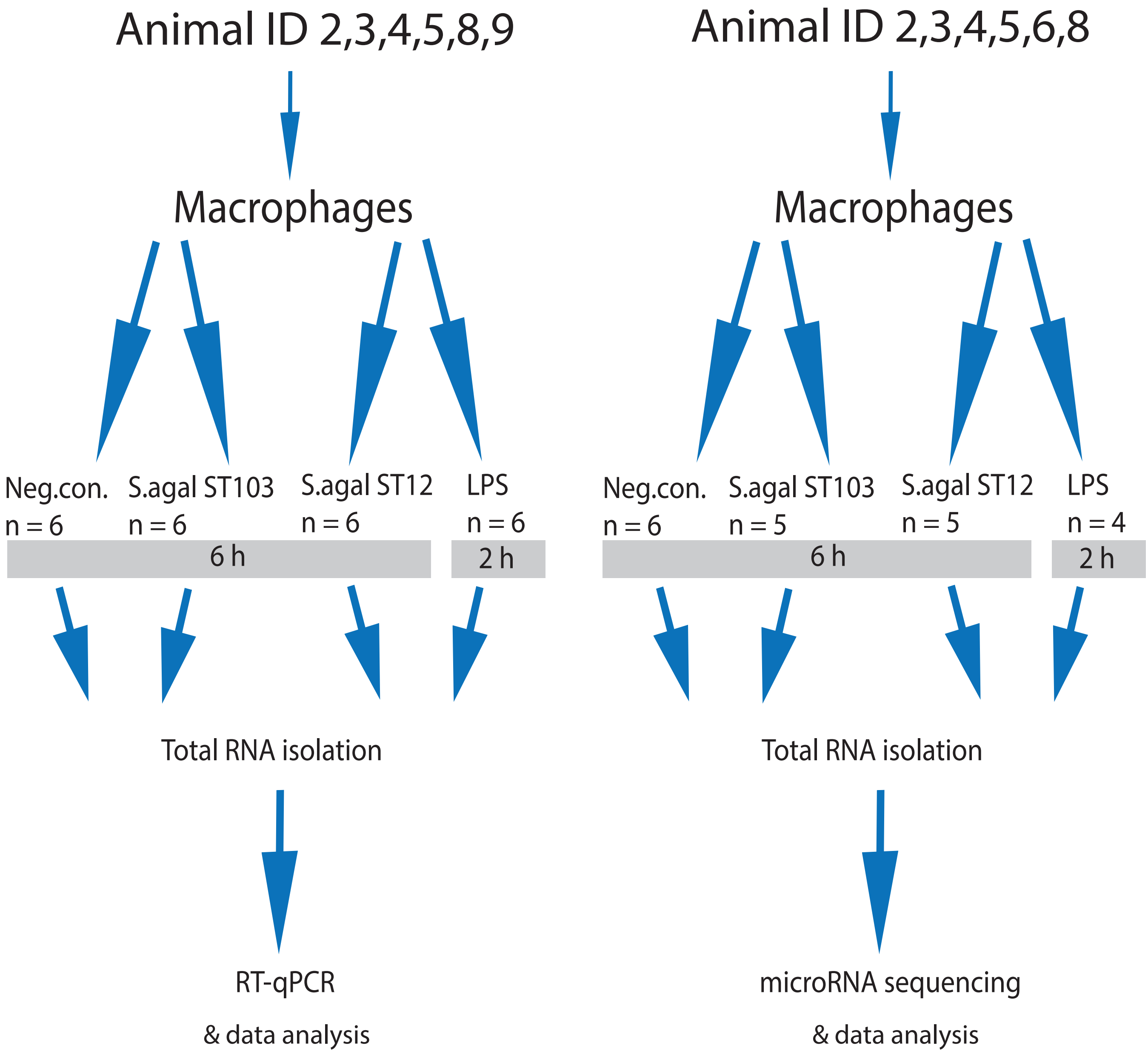
Animal ID 2,3,4,5,8,9
Macrophages
Neg.con.
n = 6
S.agal ST103
n = 6
S.agal ST12
n = 6
LPS
n = 6
6 h
2 h
Total RNA isolation
RT-qPCR
& data analysis
Animal ID 2,3,4,5,6,8
Macrophages
Neg.con.
n = 6
S.agal ST103
n = 5
S.agal ST12
n = 5
LPS
n = 4
6 h
2 h
Total RNA isolation
microRNA sequencing
& data analysis
