# Supplementary material for: MicroRNA expression profiles of bovine monocyte-derived macrophages infected in vitro with two strains of Streptococcus agalactiae
Source: BMC Genomics. 2018 Apr 10;19:241. doi: 10.1186/s12864-018-4591-3 (PMC5894239; doi:10.1186/s12864-018-4591-3)
Supplement: Supplementary file 3 — Table S2. List of primers used for reverse transcription-quantitative PCR (RT-qPCR). (DOCX 14 kb) [file 12864_2018_4591_MOESM3_ESM.docx]

**Table S2.**

List of primers used for reverse transcription-quantitative PCR (RT-qPCR).

| Gene symbol, accession no. | Primers (5’🡪3’) | Amplicon (bp) | Reference |
| --- | --- | --- | --- |
| TNFα, NM_173966.3  *tumor necrosis factor alpha* | TCTTCTCAAGCCTCAAGTAACAAG  CCATGAGGGCATTGGCATAC | 103 | Lewandowska-Sabat *et al*. BMC Genomics, 2013; 14:891 |
| IL1β, NM_174093.1  *interleukin-1 beta* | AAAAATCCCTGGTGCTGGCT  CATGCAGAACACCACTTCTCG | 89 | This work |
| IL-6, NM_173923.2  *interleukin-6* | CCTGAAGCAAAAGATCGCAGA  TGCGTTCTTTACCCACTCGT | 97 | This work |
| IL-8, NM_173925.2 *interleukin-8* | AACGAGGTCTGCCTAAACCC TGCTTCTCAGCTCTCTTCACAA | 77 | This work |
| IL-10, NM_174088.1  *interleukin-10* | TATCCACTTGCCAACCAGCC  GGCAACCCAGGTAACCCTTA | 152 | This work |
| TGFβ1, NM_001166068.1  *Transforming growth factor beta 1* | CAATTCCTGGCGCTACCTCA  GCCCTCTATTTCCTCTCTGCG | 121 | This work |
