# Supplementary figures and images for: MicroRNA expression profiles of bovine monocyte-derived macrophages infected in vitro with two strains of Streptococcus agalactiae
Source: BMC Genomics. 2018 Apr 10;19:241. doi: 10.1186/s12864-018-4591-3 (PMC5894239; doi:10.1186/s12864-018-4591-3)

a

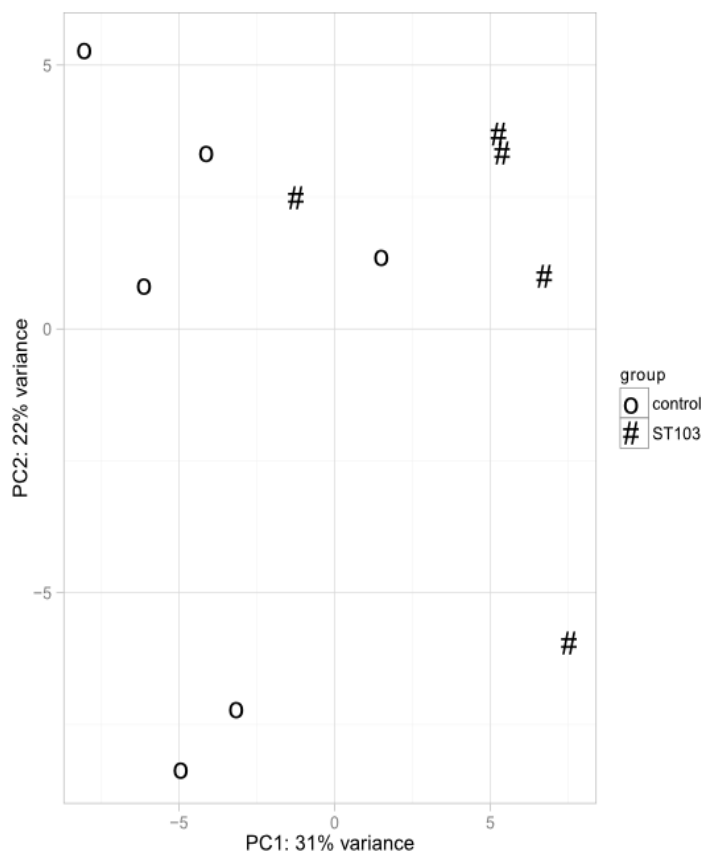

b

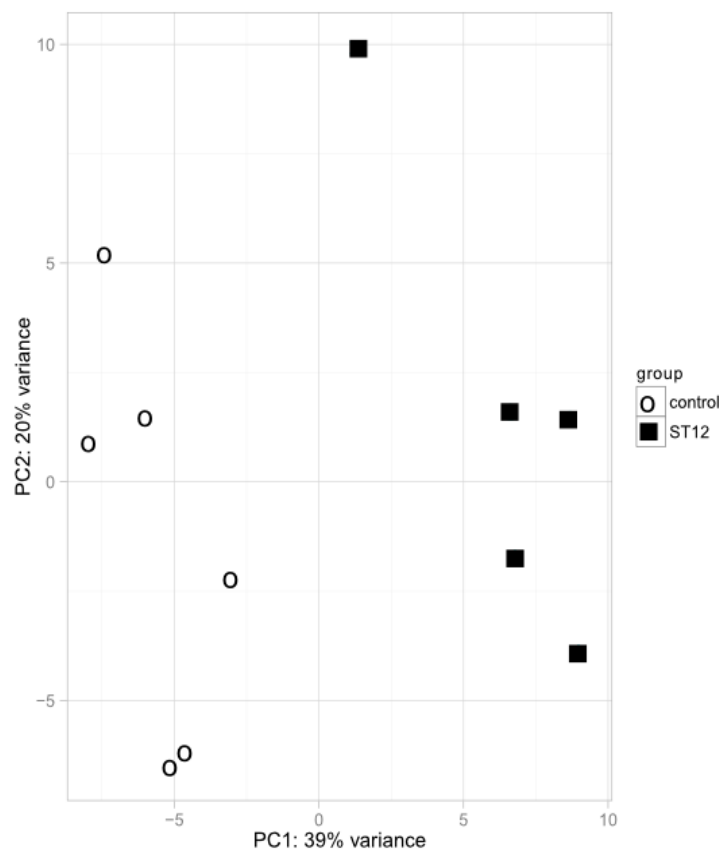

c

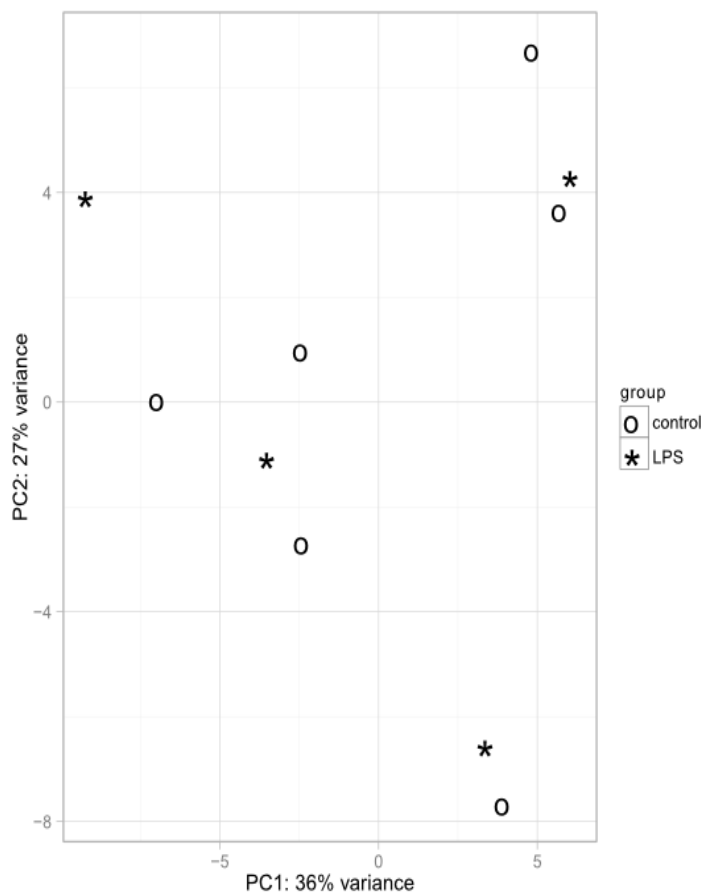

d

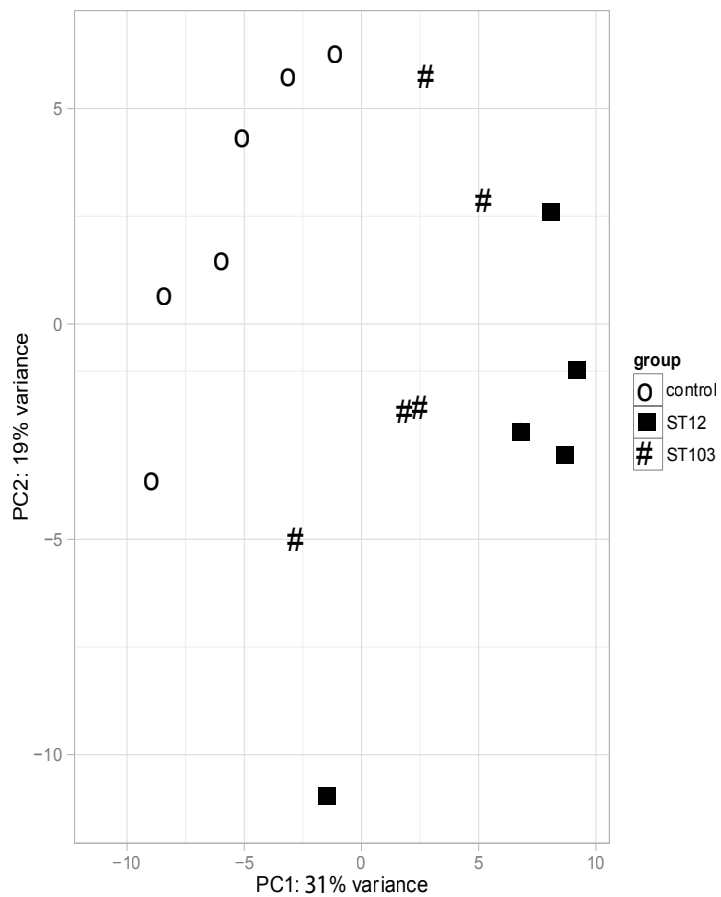

Supplement: Supplementary file 6 — Figure S2. Principal component analysis of mapped sequence reads. Control represents sequences from non-infected bovine monocyte-derived macrophage libraries, and a) Streptococcus agalactiae strain ST103 infected; b) Streptococcus agalactiae strain ST12 infected; c) LPS-challenged; and d) Streptococcus agalactiae strain ST103 or strain ST12 infected macrophages isolated from the same animals. (PDF 264 kb) [file 12864_2018_4591_MOESM6_ESM.pdf]
